# Unique ECG Findings in Acute Pulmonary Embolism: STE with Reciprocal Changes and Pathologic Q Wave

**DOI:** 10.1155/2018/7865894

**Published:** 2018-04-03

**Authors:** Amanda Grant-Orser, Brennan Ballantyne, Wael Haddara

**Affiliations:** ^1^Department of Medicine, Schulich School of Medicine, Western University, London, ON, Canada; ^2^Division of Cardiology, Department of Medicine, Schulich School of Medicine, Western University, London, ON, Canada; ^3^Division of Critical Care Medicine and Division of Endocrinology & Metabolism, Department of Medicine, Schulich School of Medicine, Western University, London, ON, Canada

## Abstract

A 68-year-old male presented to the emergency department with retrosternal chest pain, presyncope, and then a pulseless electrical activity cardiac arrest. An ECG prior to his arrest revealed ST elevations in leads V1–V3, Q waves in lead V2, and reciprocal ST depressions in the lateral and inferior leads. He received thrombolytic therapy for a presumptive diagnosis of ST elevation myocardial infarction. Return of spontaneous circulation was achieved and he underwent a coronary angiogram. No critical disease was found and his left ventriculogram showed normal contraction. His ongoing metabolic acidosis and dependence on an intra-aortic balloon pump, despite adequate cardiac output, prompted a CT pulmonary angiogram which showed multiple segmental filling defects. He was treated for a pulmonary embolism and was discharged 5 days later. Acute pulmonary embolism (APE) has variable clinical presentations. To our knowledge, this is the first case report of an APE presenting with these ECG findings suggestive of myocardial ischemia. In this case report, we discuss the underlying physiological mechanisms responsible and offer management suggestions for emergency department and critical care physicians to better expedite the treatment of APE mimicking acute coronary syndrome on ECG.

## 1. Introduction

Acute pulmonary embolism (APE) has variable clinical manifestations and should always be on a differential for shortness of breath, chest pain, or syncope. The ECG remains one of the first and most widely used tools in many work-ups due to its convenience, availability, and cost.

## 2. Case Presentation

**Figure 1:**
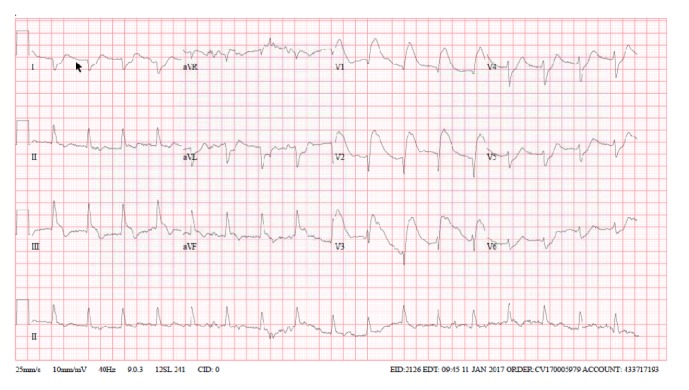
Acute pulmonary embolism presenting with STEMI-like ECG changes.

Mr. A, a 68-year-old male with no prior medical history, called EMS after experiencing retrosternal chest pain and presyncope. He subsequently collapsed and had a pulseless electrical activity (PEA) cardiac arrest. He was initially stabilized and a 12-lead electrocardiogram (ECG) was performed (Figure 1) that revealed ST elevations (STE) in leads V1–V3, Q waves in lead V2, and reciprocal ST depressions in lateral and inferior leads. He had a second PEA arrest for which he received CPR and thrombolytic therapy for a presumptive diagnosis of STE myocardial infarction (MI). Return of spontaneous circulation was achieved. Mr. A was transferred to the cardiac catheterization suite for angiography which showed no hemodynamically significant stenosis or evidence of disrupted plaque in any arteries. The left ventriculogram showed normal contraction of all segments with no dissection, left ventricular aneurysm, or mitral regurgitation. An intra-aortic balloon pump (IABP) was positioned to support the patient's hypotension. Given his normal angiogram yet ongoing metabolic acidosis and dependence on IABP despite adequate cardiac output, a pulmonary embolism was deemed the most likely aetiology. A CT pulmonary angiogram showed multiple segmental filling defects within the pulmonary arteries of the left lobe consistent with acute pulmonary embolism (APE), and the patient was treated accordingly. He was discharged from hospital five days later on room air at his functional baseline.

## 3. Discussion

The majority of clinically relevant APEs have ECG changes at presentation in the ED [1–4]. A recent meta-analysis of over 8,000 patients reported sinus tachycardia, T-wave inversions in lead V1, and ST elevations in aVR to be the most frequent abnormal ECG findings. The classically taught “McGinn-White Sign” (S1Q3T3) was seen in 24% of cases [3]. Right axis deviation is also characteristic of APE and is represented by negative T waves in the inferior and precordial leads [5]. ECG findings also have prognostic utility in APE with findings of right heart strain and atrial arrhythmias portending a worse prognosis [2, 3, 6]. There is a paucity of data on STEs, apart from aVR elevation, in the setting of APE. A 2001 case report by Falterman et al. introduced ST elevation in the anterior leads as a rare ECG manifestation of APE [7]. Of the 12 reported cases of STE in APE to date [8–10], none had a pathologic Q wave. All but one of these cases went for coronary catheterization for presumed STEMI. The in-hospital mortality rate was 16.7%, suggesting a poorer prognosis with a STE ischemic ECG pattern, consistent with findings from Kukla et al. who found that ischemic patterns (T-wave inversion in inferior and anterior leads) are associated with higher risk of complications and mortality [11]. Earlier anticoagulation has been shown to reduce morbidity and mortality in APE and should therefore be a priority [12].

The physiologic consequences of APE which culminate in STE begin with the accumulation of inflammatory mediators which cause vasoconstriction of the pulmonary vasculature, increasing pulmonary vascular resistance [13]. RV pressure overload due to clot burden leads to acute RV systolic failure, changes to the geometry of both right and left ventricles, and eventual ventricular dyssynchrony. Left-sided pump failure develops as a consequence of low LV preload and cardiac output falls [14]. Paradoxical embolism through right-to-left shunts has also been hypothesized. More recently, possible roles of catecholamine and histamine-mediated cellular ischemia and microcirculatory dysfunction have also been proposed [15, 16].

To our knowledge, this is the first case of APE presenting with the ischemic ECG changes of profound STE with a concave slope in V1–3 and reciprocal inferior-lateral ST/T inversions in I, avL, V5-6 and II, III, avF, and a rare pathological Q wave in V2. Together these findings are a poor prognostic marker for APE, particularly the QR sign in V1 and STE in V1–3 [17].

This STEMI-like presentation poses a dilemma for diagnosis and treatment. Due to the higher frequency of ACS causing STE on ECG, most patients will undergo PCI first, with a delay in imaging for APE. However, our case illustrates that APE can mimic ECG changes of STEMI more closely than previously observed. Previous studies have shown strong diagnostic accuracy when using bedside ultrasound in the initial evaluation of critically ill patients [18]. We advocate that, in situations where the diagnosis of STEMI is in question, such as an unconvincing clinical story, profound hypoxia in the absence of pulmonary edema, PEA arrest, or clinical signs of right-sided heart failure, bedside ultrasound be employed to evaluate for RV overload or failure suggestive of APE versus wall motion abnormalities more suggestive of acute coronary syndrome.
